# Towards Automatic Crack Size Estimation with iFEM for Structural Health Monitoring

**DOI:** 10.3390/s23073406

**Published:** 2023-03-23

**Authors:** Daniele Oboe, Dario Poloni, Claudio Sbarufatti, Marco Giglio

**Affiliations:** Politecnico di Milano, Mechanical Engineering Department, Via La Masa 1, 20156 Milano, Italy; daniele.oboe@polimi.it (D.O.); dario.poloni@polimi.it (D.P.); marco.giglio@polimi.it (M.G.)

**Keywords:** inverse finite element method (iFEM), digital twin, structural health monitoring, crack, digital image correlation

## Abstract

The inverse finite element method (iFEM) is a model-based technique to compute the displacement (and then the strain) field of a structure from strain measurements and a geometrical discretization of the same. Different literature works exploit the error between the numerically reconstructed strains and the experimental measurements to perform damage identification in a structural health monitoring framework. However, only damage detection and localization are performed, without attempting a proper damage size estimation. The latter could be based on machine learning techniques; however, an a priori definition of the damage conditions would be required. To overcome these limitations, the present work proposes a new approach in which the damage is systematically introduced in the iFEM model to minimize its discrepancy with respect to the physical structure. This is performed with a maximum likelihood estimation framework, where the most accurate damage scenario is selected among a series of different models. The proposed approach was experimentally verified on an aluminum plate subjected to fatigue crack propagation, which enables the creation of a digital twin of the structure itself. The strain field fed to the iFEM routine was experimentally measured with an optical backscatter reflectometry fiber and the methodology was validated with independent observations of lasers and the digital image correlation.

## 1. Introduction

Structural integrity and damage assessment are crucial factors to guarantee the safety of different structures and components. Safety requirements depend on the particular application and are defined in relation to the potential consequences induced by failure or a malfunction. Aeronautical structures are particularly critical from the safety point of view since failure can induce catastrophic consequences. For this reason, non-destructive controls (such as visual inspection, liquid penetrant, radiography, magnetic particles, etc.) are performed at scheduled intervals to detect the presence of damage early. However, these controls frequently require service interruption with a significant economic impact on the overall aircraft management. This problem is mitigated by the adoption of structural health monitoring (SHM) and prognostic health monitoring (PHM) techniques, which aim to perform a real-time assessment of the structure. The aircraft is equipped with permanently installed sensors able to represent the working conditions; then, data-driven [1,2,3] or model-based [4,5] algorithms can early detect anomalies. This can increase the service time between two consecutive maintenances, decreasing the overall cost. Data-driven algorithms frequently rely on historical data to detect anomalies, while the latter approach is based on structure models in both the healthy and damaged states. Then, algorithms frequently exploit machine learning, e.g., artificial neural networks (ANN), and a database of damage scenarios to perform the final structure’s assessment [6]. However, one main limitation of most of the literature’s SHM techniques is their dependency on the external load applied to the structure [7], which often hampers their applicability in real scenarios.

In recent years, there was a further shift of paradigm in the structure’s monitoring with the adoption of the digital twin (DT) approach [8,9,10,11]. Digital twin is an integrated multi-physics, multi-scale, probabilistic simulation of an as-built system that uses the best available physical models, sensor updates, fleet history, etc., to mirror the life of its corresponding flying twin [12]. For each physical aeronautical structure, there is a related DT, i.e., a digital representation of the same through a high-fidelity model. The number of DT literature examples is rapidly increasing in the last years, with applications in the aeronautical field [12,13,14,15,16], marine industry (offshore wind turbine and naval transport) [17,18,19], and manufacturing processes [20,21,22]. Focusing on aeronautical applications, nowadays DTs are mainly used to model the airframe of aircraft [13,14,16] and to predict the structural fatigue life of cracked structures [18,23]. Although there is not a unique literature definition of the components of a DT [9], the following main elements can be identified: (i) a physical structure and/or system, (ii) a digital representation (*master model*) of the structure/system, (iii) a connection between the physical and the virtual structure/system, which is generally represented by sensors, often referred to as *digital shadow*. In particular, the DT model must be fast enough to run in real-time, parallelly to the operation of the real structure, based on data from sensors or simulated data to predict future states of the system. For this reason, DTs are frequently based on ANN algorithms, analytical relations, other surrogate models, or a combination of these approaches. Another fundamental aspect is that the DT must be a perfect mirror of the real structure, thus the occurrence of damage or an anomaly must be reflected in the model. This requires a constant update of the DT model, where any modification of the structure/system (also due to maintenance) is reported. In conclusion, DT allows near real-time updating of the structural model through physical measurements acquired from sensors to perform damage diagnosis and prognosis in an SHM framework.

Among the different algorithms available in the literature, the inverse Finite Element Method (iFEM) is a model-based technique to compute the structure’s displacement field from strain measurements. It requires only a mesh discretization of the structure with a definition of boundary conditions, and its efficient formulation is suitable for real-time applications. Furthermore, it does not require any material property definition or knowledge of the loading condition, only the strain measurements acquired from sensors are needed as input. More specifically, iFEM generally requires strain sensors (e.g., stain gauges or fiber optic sensors) bonded on the external sides of the structure, although applications with embedded sensors (for composite materials) are available in the literature [24]. These characteristics make the iFEM attractive for a DT and, to the best authors’ knowledge, no application has been already reported in the literature. However, one main limitation hampering the implementation of the current iFEM approach in a DT framework is the lack of model updating capability, thus this manuscript aims to cover this literature gap in view of future DT applications. In particular, current iFEM applications are always based on an initial model definition (in the undamaged configuration), without any updating to account for damage propagation or maintenance operations. This paper newly proposes an updating framework for damage identification with the iFEM, which paves the way for DT practioners to employ iFEM models rather than direct methods such as FEM and XFEM, where damage introduction and propagation are already state-of-the-art. This is on the basis that the application of the latter methods becomes significantly challenging for applications subjected to unknown and stochastic loading conditions where only strain measurements are available, which justifies the development of the inverse FEM framework.

The iFEM was originally introduced by A. Tessler et al. [25,26] and nowadays its formulation is available for beam [27,28,29,30,31,32,33] and shell structures [34,35,36,37,38,39,40,41]. It is based on the minimization of a least-square functional, which is representative of the error between the input strain measurements acquired by sensors and a numerical formulation of the same function of the unknown nodal degrees of freedom (dof). Focusing on shell structures, three types of inverse elements are currently available in the literature for mesh discretization: (i) the iMIN3, a triangular element based on bilinear anisoparametric shape functions [42,43], (ii) the iQS4, a quadrilateral flat element based on bilinear anisoparametric shape functions [37,44,45], and (iii) the iCS8, an eight-nodes curved element based on quadratic isoparametric shape functions [46]. A comparative study assessed the performance of these elements also in relation to the different application scenarios [47]. In addition to the basic elements described, additional formulations have been introduced by the different authors to increase the results’ accuracy of specific classes of problems. In particular, the refined zig-zag theory (RZT) is introduced in the iFEM formulation to model the through-the-thickness displacement field of composite laminate [48,49,50,51,52], while iFEM isogeometric analysis is beneficial in the case of large non-linear deformations [53].

Several shape sensing applications with iFEM are available in the literature, based both on numerical and experimental case studies. However, although strain sensors can be applied to the whole structure when dealing with numerical case studies [44,54], their number and locations are one of the main constraints for practical applications [39,55]. Hardware limitations frequently limit the number of sensors available and their installation is subordinated to practical reasons, such as access to the structure. As a consequence, sensors generally cover only a limited portion of the structure and their location must be optimized, according to the different constraints, to well describe the strain field. Furthermore, the adoption of pre-extrapolation techniques has been revealed as an effective tool to increase the overall results’ accuracy, providing an input strain value also where sensors are not available [24,56]. In particular, this can be based on data-driven approaches, such as polynomial functions or smoothing element analysis (SEA) [57,58,59,60], on a physics-based approach [61], or Gaussian process interpolation for a statistical input strain evaluation [62]. More specifically, data-driven approaches pre-extrapolate the strain field only based on the measurements acquired from sensors; thus, the sensor network must be representative of the strain field within the structure to obtain accurate results. However, in the case of high strain gradients induced by local discontinuities (e.g., holes and notches), the design of a sensor network able to well describe the strain field is a challenging task, in particular when the number of sensors is limited. Thus, the adoption of a physics-based pre-extrapolation approach combines the strain measurements from the available sensors with the physical knowledge of the discontinuity (e.g., its size and position) to better pre-extrapolate the strain field. In particular, this approach relies on the analytical stress solution of the discontinuity itself, when available, otherwise on its numerical stress solution computed with FEM.

In addition to shape sensing, iFEM has been also extended to damage detection in an SHM framework with different approaches, such as load-independent damage indices [44,55], damage parameters based on the Von Mises strain [36,63], damage indices based on the pseudo-excitation approach [54], and artificial neural networks [64]. However, these approaches are able to identify the presence and, in some cases, the location of damage in the structure (both metallic and composites) but not estimate its size, which is still an unexplored area of iFEM applications. In particular, the numerical case studies proposed by Colombo et al. [44] investigate crack detection on a metallic plate with a load-independent damage index also accounting for different crack sizes and orientations. Furthermore, in this study, a rough estimation of the crack size could be performed from the damage index pattern since all the elements around the crack itself are covered by sensors. Similarly, Li et al. [54] detected and quantified delamination on a composite material specimen with a dense sensor network. However, these approaches are only feasible in a numerical case study and cannot be performed in a real scenario, where only sparse sensor networks are allowed. Another limitation of the previous literature works is the requirement of a damage index threshold to detect the presence of the damage itself, whose selection is subordinated to the specific case study. Only Kefal et al. recently proposed a coupling of periodynamics analysis and iFEM for crack monitoring in composite plate structures [65]; however, an automatic crack size estimation through the iFEM remains unexplored. For this reason, this work aims to define an automatic routine to perform damage size estimation in a realistic scenario within the iFEM approach. In particular, the damage is systematically introduced in the iFEM model creating different possible damage scenarios, then a maximum likelihood estimation framework selects the model that better describes the experimental strain field measured by test sensors. Thus, the damage is introduced in the iFEM model better approaching the actual condition of the physical structure, which could be used for future DT frameworks. The approach proposed fundamentally differs from the ones available in the literature since it includes the damage in the iFEM model and updates it, rather than relying on postprocessing of the strain and displacement output of the iFEM. We believe the approach presented in this work to be more promising and more robust, despite being more computationally expensive. The methodology is experimentally verified on an aluminum plate subjected to fatigue crack propagation. The strain field is experimentally measured by an optical backscatter reflectometry fiber and from independent observations of the digital image correlation, which is used to further validate the methodology.

The manuscript is organized as follows. A review of the iFEM methodology is reported in Section 2, while its extension for crack size estimation is provided in Section 3. The experimental case study is presented in Section 4 and the results and discussion are provided in Section 5. Finally, the conclusions of the work are stated in Section 6.

## 2. Inverse Finite Element Method Review

An overview of the iFEM [25,26] is reported in this section, while a more detailed review specific to the iQS4 element is available in [37,44] for interested readers.

Suppose a shell structure is discretized into finite elements, as in the direct FEM. However, the mesh is composed of inverse elements, the iQS4 in this case, which computes the displacement field from input strain measurements. This is carried out by minimizing the least-square functional of Equation (1), which is defined as the error between the input strain field acquired by sensors $\left(\cdot^{\varepsilon }\right)$ and its numerical formulation (⋅(u)), which is in turn a function of the unknown nodal displacements u. Both the input and the numerical strain fields are decoupled into three main components: the membrane e, the bending k, and the transverse shear g strain contributions. Thus, the formulation of the *i*-th inverse element can be defined as:

(1) $$\Phi_{i}\left(u^{i}\right)=\|e\left(u^{i}\right)-e_{i}^{\varepsilon }\|{}_{W_{m}^{i}}^{2}+\|k\left(u^{i}\right)-k_{i}^{\varepsilon }\|{}_{W_{b}^{i}}^{2}+\|g\left(u^{i}\right)-g_{i}^{\varepsilon }\|{}_{W_{s}^{i}}^{2}$$

where ${\left\|\cdot \right\|}_{W}^{2}$ is the squared weighted Euclidean norm with the weight matrix W. In particular, $W_{m}^{i}$, $W_{b}^{i}$, and $W_{s}^{i}$ are diagonal matrices of weights for the membrane, bending, and transverse shear strain contributions, respectively. These coefficients control the coherence between the numerical and the experimental strain measurements, in particular, in the case of sparse sensor networks. In general, a unitary reference value is associated with the elements in which the input strain field component is acquired by physical sensors, while, in other cases, the coefficients are generally reduced to small values (e.g., $10^{-4}$). Notice that each matrix W contains three weights on the main diagonal, which are related to the strain components along the *x*-axis, the *y*-axis, and the in-plane shear with respect to the element’s local reference system. Then, in case an element is interested by a monoaxial strain sensor, only the weight related to its direction is assigned equal to one and the others are reduced to a small value.

After a proper assembly procedure, the unknown structure’s displacement field is computed by minimizing the error functional presented in Equation (1), which will be detailed in Section 2.3 after a proper definition of the input (Section 2.1) and numerical (Section 2.2) strain fields.

### 2.1. Input Strain Formulation

In the most general case, the input strain formulation is computed from the strain measurements acquired on the structure. Sensors are generally applied on the external surfaces of the component, where their installation and maintenance are easier, although applications with embedded sensors are also possible [24].

**Figure 1:**
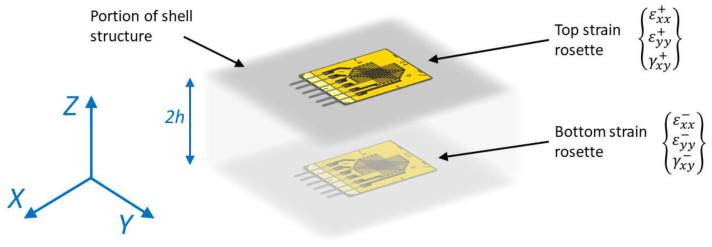
Discrete sensor location both on the top and on the bottom surface of the shell structure.

For example, consider a couple of strain gauge rosettes applied on the two external sides of the shell as shown in Figure 1. The membrane and the bending strain components associated with the *j*-th sensors’ location within the *i*-th inverse element can be defined as:

(2) $$e_{ij}^{\varepsilon }=\frac{1}{2}{\left\{\begin{array}{c} \varepsilon_{xx}^{+}+\varepsilon_{xx}^{-}\\ \varepsilon_{yy}^{+}+\varepsilon_{yy}^{-}\\ \gamma_{xy}^{+}+\gamma_{xy}^{-} \end{array}\right\}}_{j}\;\;\;\;\;\;\;\;\;\;\;\;\;\;\;\;\;\;\;\;k_{ij}^{\varepsilon }=\frac{1}{2h}{\left\{\begin{array}{c} \varepsilon_{xx}^{+}-\varepsilon_{xx}^{-}\\ \varepsilon_{yy}^{+}-\varepsilon_{yy}^{-}\\ \gamma_{xy}^{+}-\gamma_{xy}^{-} \end{array}\right\}}_{j}$$

where 2 h is the shell thickness at the sensors’ location. The computed strain components contain the information of a plane strain tensor and, in case monoaxial strain sensors are used, only one component will be defined, and the others are posed equal to zero.

Furthermore, in practical applications only a few sensors are available due to costs, space constraints, and hardware limitations, limiting the definition of the input strain field. However, for an accurate iFEM computation, the input strain field should be provided on all the structure’s inverse elements, and it should be representative of the strain gradient. For this reason, the sensor network should be properly designed, and the element’s size can be tuned according to the expected strain gradient. Nevertheless, several elements of the mesh can be free from any input definition and, to limit their influence on the global formulation, small weighting coefficients $W_{i}$ (i=m,k,s) can be associated to these elements. To further improve the iFEM results’ accuracy, the input strain field can be pre-extrapolated in the element’s locations in which physical sensors are not available, obtaining the definition of the input strain field on the whole structure. This can be carried out with different approaches according to the specific problem, such as polynomial fitting and smoothing element analysis [57,58,59,60]. These techniques are purely based on the acquired strain measurements from sensors, thus being defined as data-based approaches, only providing a more continuous and smooth strain field in the considered domain. Then, the recent introduction of the physics-based strain pre-extrapolation technique [61] allows a further increase in the iFEM results’ accuracy in the case of sparse sensor networks on notched structures. In this case, the physical knowledge of the discontinuity together with its analytical stress function allows an accurate definition of the input strain field. This pre-extrapolation technique will be detailed in Section 3.4.2 for the particular case under analysis, i.e., a cracked plate.

### 2.2. Numerical Strain Formulation

The numerical strain formulation required by Equation (1) is based on the element’s shape functions as in any finite element approach.

**Figure 2:**
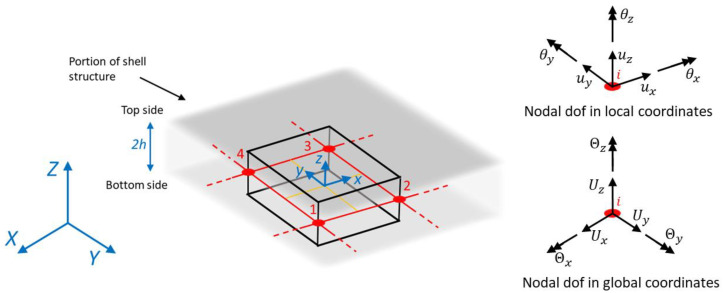
iQS4 element with global (X,Y,Z) and local (x,y,z) reference systems and the related degrees of freedoms (dof). Numbers 1 to 4 refer to the element nodes.

A local reference system (x,y,z) is defined within each inverse element, with its origin in the centroid of the element and with the *z*-axis defining the out-of-plane coordinate, so that z∈[−h;+h], as also illustrated in Figure 2. The local coordinates are computed from the global reference system (X, Y, Z), in which the structure is defined, with a proper transformation matrix, specific for each element.

Each iQS4 inverse element is composed of four nodes, each one with six degrees of freedom (dof). In particular, each element has 24 dof with 3 translations $u_{q}={\left\{u_{x},\;u_{y},\;u_{z}\right\}}^{q}$ and 3 rotations $\theta_{q}={\left\{\theta_{x},\;\theta_{y},\theta_{z}\right\}}^{q}$ for each element’s node q=(1,2,3,4), which are collected into the element’s nodal displacement vector $u^{i}$. Then, the local displacement field within each inverse element is defined through the shape functions $N_{q}$, $L_{q}$, and $M_{q}$ [37] as:

(3) $$u\left(x,y\right)=\sum_{q=1}^{4}N_{q}u_{xq}+\sum_{q=1}^{4}L_{q}\theta_{zq}\;v\left(x,y\right)=\sum_{q=1}^{4}N_{q}u_{yq}+\sum_{q=1}^{4}M_{q}\theta_{zq}\;w\left(x,y\right)=\sum_{q=1}^{4}N_{q}u_{zq}-\sum_{q=1}^{4}L_{q}\theta_{xq}-\sum_{q=1}^{4}M_{q}\theta_{yq}\;\theta_{x}\left(x,y\right)=\sum_{q=1}^{4}N_{q}\theta_{xq}\;\theta_{y}\left(x,y\right)=\sum_{q=1}^{4}N_{q}\theta_{yq}$$

Then, under the hypothesis of plane stress condition and after computing the partial derivatives of the shape functions, the strain field components within each element can be defined as:

(4) $$e\left(u^{i}\right)=B^{m}u^{i}\;k\left(u^{i}\right)=B^{b}u^{i}\;g\left(u^{i}\right)=B^{s}u^{i}$$

where $B^{m}$, $B^{b}$, and $B^{s}$ are matrices containing the derivatives of the shape functions.

Finally, the numerical strain field can be computed with the following relations in correspondence of the required z coordinate:

(5) $$\left\{\begin{array}{c} \varepsilon_{xx}\\ \varepsilon_{yy}\\ \gamma_{xy} \end{array}\right\}=e\left(u^{i}\right)+z\cdot k\left(u^{i}\right)\;\left\{\begin{array}{c} \gamma_{xz}\\ \gamma_{yz} \end{array}\right\}=g\left(u^{i}\right)$$

### 2.3. Matrix Formulation

As previously introduced, the iFEM relies on the minimization of the global formulation of Equation (1); however, it was limited to a single element until now. Thus, before considering the contribution of all the inverse elements discretizing the structure, Equation (1) is elaborated to achieve an efficient numerical computation. In particular, the 3 square weighted norms are expressed as normalized Euclidean norms as:

(6) $$\|e\left(u^{i}\right)-e_{i}^{\varepsilon }\|{}_{W_{m}^{i}}^{2}=\frac{1}{n}\iint_{A_{i}}^{}\overset{n}{\underset{j=1}{\sum }}{\left(e{\left(u^{i}\right)}_{j}-e_{ij}^{\varepsilon }\right)}^{T}W_{m}^{i}\left(e{\left(u^{i}\right)}_{j}-e_{ij}^{\varepsilon }\right)dxdy\;\|k\left(u^{i}\right)-k_{i}^{\varepsilon }\|{}_{W_{b}^{i}}^{2}=\frac{{\left(2h\right)}^{2}}{n}\iint_{A_{i}}^{}\overset{n}{\underset{j=1}{\sum }}{\left(k{\left(u^{i}\right)}_{j}-k_{ij}^{\varepsilon }\right)}^{T}W_{b}^{i}\left(k{\left(u^{i}\right)}_{j}-k_{ij}^{\varepsilon }\right)dxdy\;\|g\left(u^{i}\right)-g_{i}^{\varepsilon }\|{}_{W_{s}^{i}}^{2}=\frac{1}{n}\iint_{A_{i}}^{}\overset{n}{\underset{j=1}{\sum }}{\left(g{\left(u^{i}\right)}_{j}-g_{ij}^{\varepsilon }\right)}^{T}W_{s}^{i}\left(g{\left(u^{i}\right)}_{j}-g_{ij}^{\varepsilon }\right)dxdy$$

where $A_{i}$ is the area of the considered element and n the total number of input strain sensors within the same. Then, after substituting the input and the numerical strain components previously developed in Section 2.1 and Section 2.2, respectively, the least-square functional can be expressed as:

(7) $$\Phi_{i}\left(u^{i}\right)=u^{i^{T}}k^{i}u^{i}-2u^{i^{T}}f^{i}+\xi^{i}$$

In which:

(8) $$k^{i}=\iint_{A_{i}}^{}\left(B^{m^{T}}W_{m}B^{m}+{\left(2h\right)}^{2}B^{b^{T}}W_{b}B^{b}+B^{s^{T}}W_{s}B^{s}\right)dxdy\;f^{i}=\frac{1}{n}\iint_{A_{i}}^{}\overset{n}{\underset{j=1}{\sum }}\left(B^{m^{T}}W_{m}e_{ij}^{\varepsilon }+{\left(2h\right)}^{2}B^{b^{T}}W_{b}k_{ij}^{\varepsilon }+B^{s^{T}}W_{s}g_{ij}^{\varepsilon }\right)dxdy\;\xi^{i}=\frac{1}{n}\iint_{A_{i}}^{}\overset{n}{\underset{j=1}{\sum }}\left(e_{ij}^{\varepsilon^{T}}W_{m}e_{ij}^{\varepsilon }+{\left(2h\right)}^{2}k_{ij}^{\varepsilon^{T}}W_{b}k_{ij}^{\varepsilon }+g_{ij}^{\varepsilon^{T}}W_{s}g_{ij}^{\varepsilon }\right)dxdy$$

Integrals can be efficiently computed numerically with the Gauss quadrature, for example adopting four integration points for each inverse element for an accurate result. Then, a standard assembly procedure accounts for the contribution of all structures’ inverse elements, obtaining a global formulation of Equation (7). Finally, this is minimized with respect to the global displacement field (i.e., ∂Φ/∂U=0) and thus the problem can be written as:

(9) KU=F

where K is a matrix linking the nodal displacement U, in global coordinates ($U_{x}$, $U_{y}$, $U_{z}$, $\Theta_{x}$, $\Theta_{y}$, and $\Theta_{z}$ for each node), with the vector F, which is a function of the input strain field. Notice that the matrix K depends on the structure’s element discretization and on the sensor network configuration, while F only depends on the input strain measurements. However, the matrix K is singular and it will lead to a rigid motion of the structure if unconstrained; thus, after the definition of problem-specific boundary conditions, the unconstrained (free) nodal displacements can be computed as:

(10) $$K_{FF}U_{F}=F_{F}\;\;\;\;\;\;\;\;\;\;⟹\;\;\;\;\;\;\;\;\;\;U_{F}=K_{FF}^{-1}F_{F}$$

Finally, after the computation of the structure’s displacement field, the numerical strain can be computed on the whole structure through Equation (5).

## 3. Crack Size Estimation Technique

A basic requirement of any iFEM application is that the model well describes the physical structure, which is generally true in the healthy condition of the structure. Then, in case a damage nucleates and propagates in the structure, the iFEM displacement and strain reconstruction will be different from the acquired data (often passed as input to the iFEM) due to geometrical non-compatibility. This is exploited by different literature research [36,44,55,63], where the strain difference between the acquired data and the iFEM reconstruction highlights the presence of potential damage. However, these approaches are limited to damage detection and localization, without attempting a proper diagnosis. Thus, in this section, a new strategy will be proposed to automatically estimate the damage size, in this case, the crack length on a metallic plate. In particular, the proposed approach does not require any training process and an a priori creation of a damage scenarios database for comparison is not mandatory.

### 3.1. Model Updating Procedure Overview for Damage Quantification

As already anticipated, if the iFEM model is geometrically different from the physical structure, due to damage, the displacement and strain field reconstructions will also be different from the acquired data. Thus, the idea is to systematically introduce the damage into the iFEM model to reduce its discrepancy with respect to the damaged structure. Then, the model that presents the lower discrepancy with respect to the experimental observations is the best description of the actual structural condition (e.g., its damaged state), specifically for the current mesh discretization. This approach is based on the following fundamental requirements:

- The damage must be modelled in the iFEM, which may require different levels of difficulty according to the specific damage mechanism investigated.
- The strain measurements acquired by the sensors must be sensitive to the presence of potential damages, otherwise, only the shape sensing phase can be performed. Then, sensors are split into an input and a test sensor network, as will be better specified in the following sections. In the case of sparse sensor networks, strain pre-extrapolation techniques can be adopted to improve the solution.

Finally, a major constraint of the methodology is related to the higher computation time required with respect to the basic iFEM formulation, as will be investigated in detail in the following sections.

The approach hereby introduced is in principle general and it will be detailed for the particular case of a cracked metallic plate. In particular, the crack size estimation approach presented, with being the first application of this methodology, relies on the following additional assumptions that can be gradually relieved with future developments of the methodology:

- The crack location is supposed to be exactly known from other localization approaches (manual or automatic). This hypothesis is made to not introduce additional uncertainties on the crack location and thus focus the attention on the quantification phase, with being it the main novelty. The joint estimation of the crack location, orientation, and size is left for future research.
- Only a straight propagation of the crack is investigated by opening the nodes of the existing iFEM mesh defined in the undamaged configuration. This cannot take into account an arbitrary orientation of the crack or a change in the crack propagation direction. However, if the material properties are known, the iFEM might be employed to track the loading condition and the principal strain and stress directions, which might be used to refine the mesh and potentially consider different crack orientations.

### 3.2. Crack Propagation Routine

**Figure 3:**
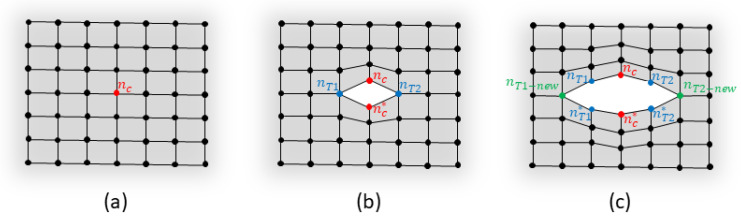
Crack propagation routine: (**a**) identification of the crack center (red node); (**b**) crack introduction; (**c**) crack propagation.

Starting from an iFEM model defined in the healthy condition, the first step regards the introduction of the damage in the model itself. For the particular case of a crack in a shell structure, this can be summarized with the following procedure (Figure 3):

1. The central position of the crack is identified as one node of the undamaged iFEM mesh, as highlighted by the red node in Figure 3a. This localization can be based on several approaches, such as visual inspection of the structure or automatic localization with a specific algorithm. The present research will not investigate any particular localization technique and the crack center is supposed to be known from any visual or automatic inspection; however, the approach described remains valid for any general localization method.
2. The crack is introduced in the mesh by doubling the node corresponding to the crack center and modifying the connectivity matrix to unlink the elements (Figure 3b). The crack is supposed to propagate under a pure mode I loading; thus, its orientation is perpendicular to the first principal stress. Finally, the two nodes identifying the crack tips are highlighted in blue in Figure 3b. This procedure, together with crack center identification, is detailed in Algorithm 1.
3. The crack is further propagated perpendicularly to the first principal stress, supposing its orientation remains constant in time. The two nodes on the crack tip’s locations are doubled and the connectivity matrix is further modified to unlink the elements, as shown in Figure 3c and detailed in Algorithm 2.
4. Repeat step 3 several times, opening one node per time, until the most likely crack length is reached according to the procedure detailed in Section 3.3.

For the sake of clarity, Figure 3 shows the mesh in a generic deformed state only for a better comprehension of the same. The mesh is always defined in the undeformed configuration of the structure and thus the doubled nodes are overlapped.

**Algorithm 1:** Crack center identification and introduction on an undamaged structure1:Identify the position (x,y,z) of the crack center2:Find the maximum principal stress direction from the sensor’s measurements3:Find the nearest node of the mesh to the crack center → center node $n_{c}={\left[x,y,z\right]}_{c}$
4:Identify the two crack tip nodes $n_{T1}={\left[x,y,z\right]}_{T1}$ and $n_{T2}={\left[x,y,z\right]}_{T2}$ as the nearest nodes to the crack center $n_{c}$ and perpendicular to the maximum principal stress direction5:Double the center node $n_{c}^{*}=n_{c}$ → update the node matrix6:Update the connectivity matrix to unlink the elements in correspondence of $n_{c}$
7:Compute the crack length: 2a= norm($n_{T1}-n_{T2}$)8:Compute and invert the iFEM K matrix

**Algorithm 2:** Crack propagation from an existing damage condition1:Identify the two nodes $n_{T11}$ and $n_{T12}$ as the nearest nodes to the crack tip $n_{T1}$ and perpendicular to the maximum principal stress direction2:Identify the new crack tip 1 between $n_{T11}$ and $n_{T12}$ as the farthest node from $n_{c}$ → $n_{T1-new}$
3:Identify the two nodes $n_{T21}$ and $n_{T22}$ as the nearest nodes to the crack tip $n_{T2}$ and perpendicular to the maximum principal stress direction4:Identify the new crack tip 2 between $n_{T21}$ and $n_{T22}$ as the farthest node from $n_{c}$ → $n_{T2-new}$
5:Double the crack tip nodes: $n_{T1}^{*}=n_{T1}$ and $n_{T2}^{*}=n_{T2}$ → update the node matrix6:Update the connectivity matrix to unlink the elements in correspondence of $n_{T1}$ and $n_{T2}$
7:Update the crack tip location: $n_{T1}=n_{T1-new}$ and $n_{T2}=n_{T2-new}$
8:Compute the crack length: 2a= norm($n_{T1}-n_{T2}$)9:Compute and invert the iFEM K matrix

### 3.3. Damage State Diagnosis

Once multiple iFEM models have been generated, the next task is identifying which model is more representative of the actual structure’s damage condition and thus, in this application, estimating the damage size. Notice that, for the sake of simplicity, the procedure described relies on a database of iFEM models containing different damage conditions (generated according to Section 3.2); however, this a priori database definition is not mandatory, as will be better discussed in Section 5.2.3.

To approach this problem, the sensors available are subdivided into two sensor networks:

- An input sensor network ($\varepsilon_{inp}$) to feed the iFEM algorithm and compute the displacement and the strain field on the whole structure
- A test sensor network ($\varepsilon_{test}$) to compare the strain field computed by the iFEM ($\varepsilon_{iFEM}$) with the experimental measurements acquired on the structure.

**Figure 4:**
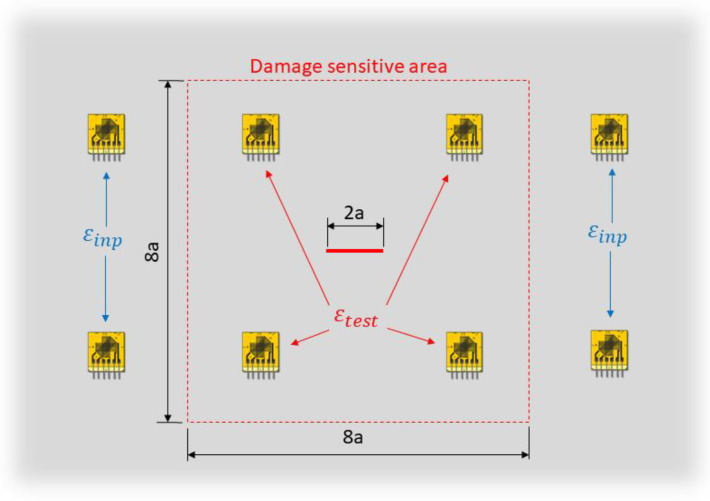
Portion of the shell structure with input and test sensor networks definition.

In the most general case, the two sensor networks can be coincident; however, to properly highlight the presence of the damage and perform a better diagnosis, the following approach is adopted (Figure 4):

- The input sensor network is composed only of sensors sufficiently far from the damaged area to acquire the far-field strain. Thus, these strain measurements will not be affected by the presence of the damage, whatever the actual damage condition considered is.
- The test sensor network is composed of sensors located in the area of influence of the damage, where the observations can be correlated to the actual damage state.

The damage-sensitive area is defined by a square domain centered on the crack location and with dimensions 8a, where a is the expected maximum semi-crack length. It should be noted that, in a general application with multiple hotspots for damage nucleation, the input and test sensor network definition can be updated after the first damage detection to maximize the performance of the algorithm. In addition, the number and the position of sensors must be tuned according to the minimum detectable damage size required by the application.

**Figure 5:**
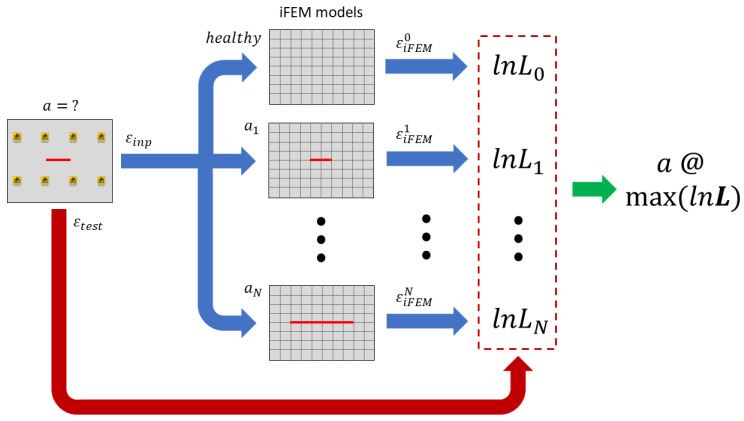
Crack size estimation framework.

Each strain pattern acquired from sensors is characterized by a specific crack length, which is unknown and the target of this analysis. Thus, for each acquisition, the input strain field is fed to all the iFEM models generated, which, in turn, will compute different displacement and strain reconstructions, as shown in Figure 5. Then, the computed strain fields will be compared to the test measurements and, in order to condensate the information from multiple sensors into a single scalar for each model, the likelihood index is adopted. In particular, for each iFEM model i, a Gaussian likelihood index is computed as:

(11) $$L_{i}=\overset{N_{test}}{\underset{j=1}{\prod }}\left(\frac{1}{\sqrt{2\pi \sigma^{2}}}\cdot \exp\left(-\frac{{\left(\varepsilon_{test}-\varepsilon_{iFEM}^{i}\right)}_{j}^{2}}{2\sigma^{2}}\right)\right)$$

where $N_{test}$ is the total number of test sensors and $\sigma^{2}$ is a variance parameter related to the measurement uncertainties.

However, with the likelihood being the product of elements smaller than one, its final value can reach very small values which cannot be correctly computed in a numerical environment, in particular when a large number of test sensors is used. Thus, to overcome this issue, logarithmic Gaussian likelihood is used:

(12) $$lnL_{i}=-\frac{N_{test}}{2}\cdot \ln\left(2\pi \sigma^{2}\right)-\frac{1}{2\sigma^{2}}\overset{N_{test}}{\underset{j=1}{\sum }}{\left(\varepsilon_{test}-\varepsilon_{iFEM}^{i}\right)}_{j}^{2}$$

The likelihood (or the logarithmic likelihood) of each model is related to its probability of well reproducing the test measurements and thus the damage state considered. Thus, the problem is addressed with a maximum likelihood estimation framework, where the iFEM model with the largest logarithmic likelihood is the one that better minimizes the error with respect to the test measurements, being the diagnosis of the actual damage state.

### 3.4. Crack Strain Field Pre-Extrapolation

The iFEM framework proposed requires a good strain reconstruction near the damage to correctly compute the damage state. However, in this framework the input strain field provides just the far-field strain, thus, although the different iFEM models contain different damage scenarios, the crack will not open and, as a consequence, it will not induce its characteristic strain field. To overcome this issue, the recently developed physics-based strain pre-extrapolation technique is adopted to impose the strain field specific of each crack length. The approach is described for the specific case of a crack in an infinite plate, while its general formulation is available in [61].

#### 3.4.1. Pre-Extrapolation Framework Overview

**Figure 6:**
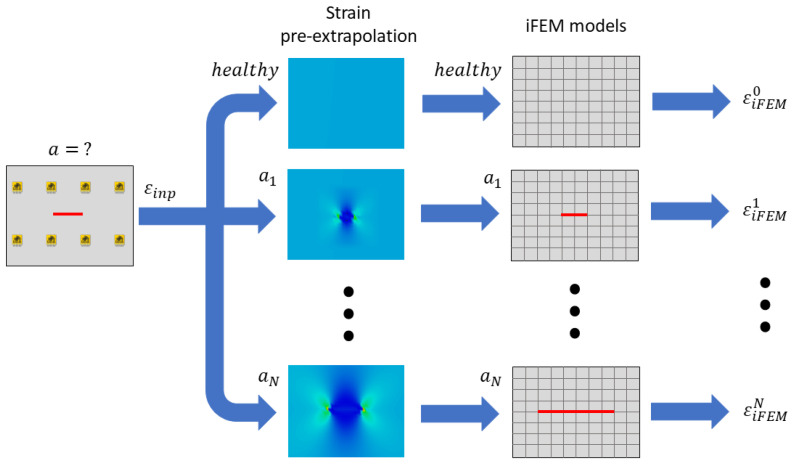
Strain pre-extrapolation framework.

As previously introduced, the physics-based strain pre-extrapolation technique is adopted to correctly compute the strain field on the whole structure and, in particular, near the crack, also in the case of a sparse sensor network. This technique accounts for the knowledge of the crack length and position, thus considering its physical strain behavior on the structure. Obviously, the correct crack scenario is not a priori known since it is the objective of the present analysis, as previously described in Section 3.3. Thus, the framework proposed (which is also summarized in Figure 5) is modified by adding the physics-based strain pre-extrapolation technique at each iteration, as detailed in Figure 6. In particular, each iFEM model considered contains a different damage scenario and thus a specific pre-extrapolated input strain field will be computed for the damage-sensitive area. Then, among all the adopted iFEM models, where each one considers a different damage scenario and its relative strain pre-extrapolation, the condition that better describes the real damage is selected with the maximum likelihood estimation framework described in Section 3.3.

#### 3.4.2. Physics-Based Strain Pre-Extrapolation for a Crack in an Infinite Plate

For each considered iFEM model (i.e., for each specific damage condition), the strain field is pre-extrapolated on the whole structure with the following physics-based approach [61]:

- A global pre-extrapolation, based on a data-driven approach, is computed on the whole structure to obtain the far-field strain value. In the present analysis, a bi-linear polynomial fitting is adopted; however, other techniques (for example the SEA) can be selected according to the specific case study. Notice that each strain component ($\varepsilon_{xx}$, $\varepsilon_{yy}$, $\gamma_{xy}$) is independently pre-extrapolated on each side of the structure.
- A local pre-extrapolation better predicts the strain field in the damage-sensitive area with a physics-based approach. This takes as input the far-field strain computed by the data-driven pre-extrapolation to compute the specific strain field induced by the crack.

In particular, the local physics-based approach is applied to a square damage-affected area with dimensions 8a (Figure 4), where a is the semi-crack length considered by the current iFEM model. The pre-extrapolation is based on the Westergaard stress solution [66], which considers a crack in an infinite plate subjected to a biaxial state of stress. However, these equations are expressed in terms of stress, while only the strain is available as input data in the iFEM formulation. For this reason, the far-field stress $\left(\sigma_{x},\;\sigma_{y},\;\tau_{xy}\right)$ is computed from the far-field strain ($\varepsilon_{xx}$, $\varepsilon_{yy}$, $\gamma_{xy}$) with the Hook’s equation under the hypothesis of plane stress condition:

(13) $$\left\{\begin{array}{c} \varepsilon_{xx}\\ \varepsilon_{yy}\\ \gamma_{xy} \end{array}\right\}=\left[\begin{array}{ccc} 1 & -\nu & 0\\ -\nu & 1 & 0\\ 0 & 0 & 2\left(1+\nu \right) \end{array}\right]\cdot \left\{\begin{array}{c} \sigma_{x}\\ \sigma_{y}\\ \tau_{xy} \end{array}\right\}$$

Notice that the elastic modulus of the material is not included in the equation since material properties are generally not required by the iFEM approach. Thus, the computed stress field is not exactly a stress, but it differs by a constant equal to the unknown elastic modulus. However, for proper computation of the stress field, the Poisson ratio ν is required and literature values can be adopted in case specific material properties are not available.

**Figure 7:**
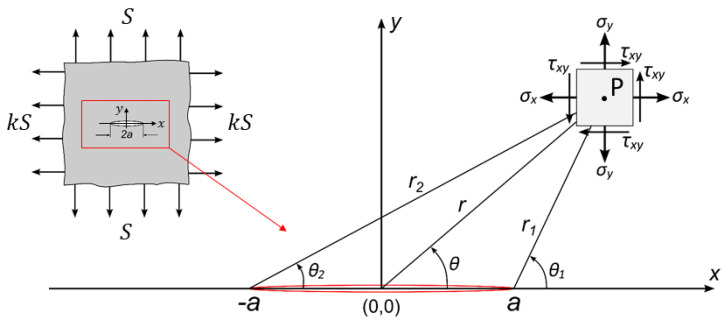
Stress field near a crack in an infinite plate subjected to a general biaxial loading condition.

Then, once the far-field stress has been computed, the Westergaard stress solution can be applied. However, its original solution accounts for biaxial far-field stress (with $\sigma_{x}=\sigma_{y}$), while only a monoaxial state of stress is considered under the hypothesis that the crack is loaded in a pure mode I. Different manipulations of the Westergaard solution are available in the literature to address specific problems; in particular, the solution proposed by Elfitis and Liebowitz [67] is reported in Equation (14). This set of equations accounts for a generic bi-axial far-field state of stress, where $\sigma_{x}=k\sigma_{y}$, as illustrated in Figure 7. In particular, the monoaxial state of stress that opens the crack in a pure mode I can be defined by imposing $S=\sigma_{y}$ and k=0. Finally, the stress field in the damage-sensitive area can be computed as:

(14) $$\sigma_{x}=\frac{S\;r}{\sqrt{r_{1}r_{2}}}\cos\left(\theta -\frac{\theta_{1}+\theta_{2}}{2}\right)-\frac{S\;a^{2}}{{\left(r_{1}r_{2}\right)}^{\frac{3}{2}}}\;r\sin\left(\theta \right)\sin\frac{3}{2}\left(\theta_{1}+\theta_{2}\right)-\left(1-k\right)S\;\sigma_{y}=\frac{S\;r}{\sqrt{r_{1}r_{2}}}\cos\left(\theta -\frac{\theta_{1}+\theta_{2}}{2}\right)+\frac{S\;a^{2}}{{\left(r_{1}r_{2}\right)}^{\frac{3}{2}}}\;r\sin\left(\theta \right)\sin\frac{3}{2}\left(\theta_{1}+\theta_{2}\right)\;\tau_{xy}=\frac{S\;a^{2}}{{\left(r_{1}r_{2}\right)}^{\frac{3}{2}}}\;r\sin\left(\theta \right)\cos\frac{3}{2}\left(\theta_{1}+\theta_{2}\right)$$

where a is the semi-crack length considered and r, $r_{1}$, $r_{2}$, θ, $\theta_{1}$, and $\theta_{2}$ are linear and angular variables defining a specific point P of the domain in which the stress field is computed, as reported in Figure 7. Notice that the input stress S is obtained from Equation (13), which is in turn a function of the strain field pre-extrapolated with a data-driven approach. In case a generic bi-axial loading condition is present, both S and K can be computed from the far-field stresses.

Finally, once the stress field induced by the crack has been computed, it can be transformed back into strains through Equation (13), defining the pre-extrapolated strain field to feed the iFEM algorithm.

## 4. Case Study

The crack size estimation methodology proposed was experimentally applied and verified on an aluminum plate subjected to a fatigue crack propagation to test its effectiveness and performance in a real scenario.

This section introduces the overall case study; in particular, the specimen with its sensor network is described in Section 4.1, the test rig with the acquisition systems is described in Section 4.2, and the iFEM models adopted are presented in Section 4.3.

### 4.1. Specimen and Sensor Network

**Figure 8:**
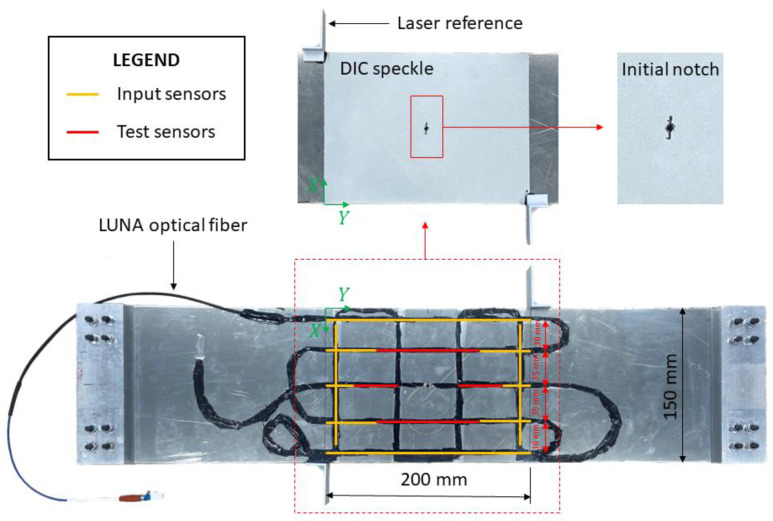
Specimen with the sensor network.

The specimen is an aluminum plate (E=70,000 MPa and ν=0.3) with overall dimensions 650×150 mm and a thickness of 2 mm (Figure 8). At the two extremities of the specimen, aluminum tabs are connected to ensure a better stress distribution into the plate once it is mounted in the testing machine. Then, an artificial notch with a total length of 12 mm is introduced in the central point of the plate to ensure proper crack nucleation and propagation during the test.

Strains are experimentally measured through a 3 m-long optical backscatter reflectometry (OBR) fiber bound on one side of the plate with the 3 M™ DP490 epoxy adhesive. The fiber pattern has been designed to cover at the best the region of interest (ROI) of the specimen, which corresponds to the central region of the plate with dimensions 200×150 mm. In particular, the dimension of the ROI has been selected to avoid any boundary effect induced by the tabs and the gripping system of the testing machine, obtaining a constant far-field stress distribution. This case study design mimics a portion of an aeronautical structure, where a crack can nucleate from a rivet hole. The fiber provides measurement points along both the x and the y axis of the plate according to the reference system in Figure 8. Then, the straight segments of the fiber inside the ROI are subdivided into the input and test sensors; in particular, input sensors are sufficiently far from the crack to acquire only the far-field strain, while test sensors are affected by the crack’s strain field.

On the other side of the specimen, a fine speckle pattern was painted to create a uniformly random texture and acquire the strain field on the whole plate’s area by the digital image correlation (DIC) technique. Finally, two reference surfaces were bonded at the two extremities of the ROI to measure its effective displacement with lasers during the test.

### 4.2. Test Rig

**Figure 9:**
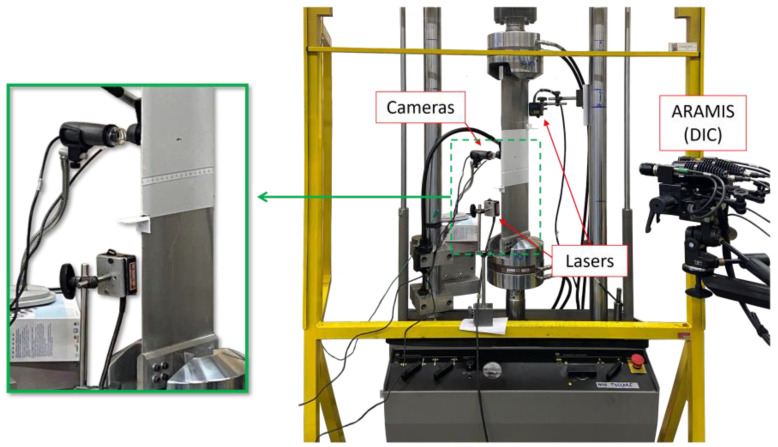
Test rig with the main acquisition systems.

The specimen was mounted on a hydraulic MTS monoaxial testing machine with a 100 kN load cell, as illustrated in Figure 9. The specimen was subjected to a tensile–tensile fatigue load with $F_{max}=20\;\mathrm{kN}$ and R=0.1 to nucleate and then propagate a crack from the artificial notch. A total number of 32,000 cycles were applied subdivided into different blocks of cycles. Then, between two consecutive blocks of cycles, data were statically acquired at the maximum load.

The OBR fiber was acquired with a LUNA ODISI-B interrogator, providing a measurement point every 2.5 mm of fiber. This fiber discretization accounts for 345 measurement points for the input sensor network and 117 measurement points for the test one. Furthermore, several strain samples were statically acquired for each crack length to average the values and reduce the noise. It has to be noticed that, since the plate is loaded in tension, the bending strain components are zero (or negligibly small) and only the membrane components contribute to the displacement field of the structure. For this reason, although sensors are applied only on one side of the structure, the membrane strain components can be correctly computed.

The crack length was measured with two Dino-Lite microscopes (1280×1024 pixel resolution) on one side of the specimen (one at each crack tip), while, on the other side of the specimen, the dual-camera ARAMIS system provided by GOM acquired the images for the DIC technique. The ARAMIS system includes 12 megapixel cameras and a dual-led lights device. The system was calibrated to acquire the images on a sub-portion of the ROI with dimensions 140×110 mm, corresponding to the maximum area available for this system. Finally, two MEL M5L/10 lasers acquire the effective displacement of the ROI. These acquisition systems (cameras and lasers) are not available in a general real SHM application and they are used just for validation in the present study; they are not used by the iFEM method presented in this study.

### 4.3. iFEM Models

**Figure 10:**
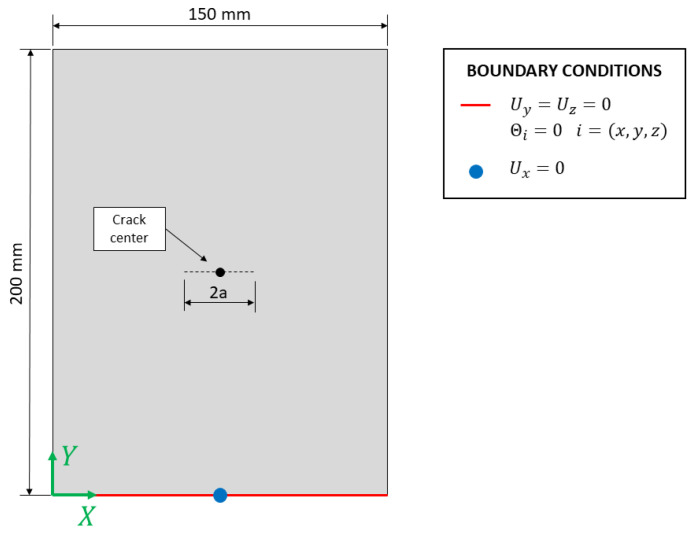
iFEM model with boundary conditions and the crack center positions.

The structure’s iFEM model is limited to the ROI, thus to a shell structure with in-plane dimensions 150×200 mm and a thickness of 2 mm, as reported in Figure 10. The boundary conditions are representative of the portion of plate in tension; in particular, the lower side of the plate is constrained to avoid any free motion in the 3D space. The horizontal displacement $U_{x}$ is constrained just in one node to allow the transversal shrinking of the plate itself.

Then, the structure is discretized into inverse elements (the iQS4) with two different structured meshes to investigate the sensitivity of the methodology to the element’s size:

- A mesh with 3.03×3 mm element’s size (namely a 3 mm model); thus, the structure is discretized by a total number of 3300 inverse elements;
- A mesh with 1×1 mm element’s size (namely 1 mm model); thus, the structure is discretized by a total number of 30,000 inverse elements.

Notice that, although only the membrane response of the element is activated, the selection of the iQS4 element is beneficial thanks to its drilling dof $\theta_{z}$ which can avoid shear locking issues near the crack.

**Figure 11:**
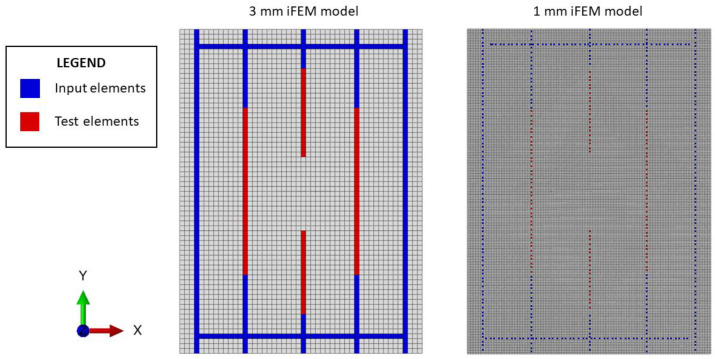
Mesh discretization (3 mm and 1 mm meshes) with input and test sensor networks.

For both cases, the structure is initially discretized in the undamaged configuration, so without the presence of any crack, and the input and the test sensor networks are defined according to Section 4.1. Notice that, since the fiber optic spatial discretization provides a measurement point every 2.5 mm, the 3 mm model contains at least one measurement in every element interested by the fiber. On the contrary, for the 1 mm model, only one element every two/three elements is covered by an experimental strain measurement, as also illustrated in Figure 11.

The central point of the crack is then identified on the structure by the node with coordinates (x, y)=(75 mm, 100 mm), which, in the present work, is assumed known a priori. Then, multiple iFEM models are generated considering different damage conditions, as previously described in Section 3. The crack is artificially increased symmetrically along the X axis with respect to the crack center since the maximum principal stress acts along the Y direction. Notice that the step increment between two consecutive crack lengths is equal to two times the element’s size considered by each model. For example, considering the 3 mm model, the following crack lengths are considered: 6, 12, 18, 24, 30, 36, 42, and 48 mm, while for the 1 mm model, the step increment is reduced to 2 mm and thus the following crack lengths are generated: 2, 4, 6, 8, …, 46, and 48 mm. Notice that, this a priori generation of the damage conditions is not strictly necessary for the algorithm’s routine since only the required models can be generated on-demand when needed, as will be better discussed in Section 5.2.3.

## 5. Results and Discussion

The data experimentally acquired in the case study under analysis (Section 5.1) were systematically analyzed to evaluate the effectiveness and the performance of the proposed crack size estimation technique. In particular, the results obtained with the 3 mm iFEM model are analyzed in detail in Section 5.2, investigating all the aspects of the technique proposed, while a sensitivity analysis on the mesh size is performed in Section 5.3 including results for the 1 mm mesh model.

### 5.1. Fatigue Propagation Experimental Results

The specimen was tested with a total number of 32,000 fatigue cycles subdivided into 10 different blocks. At the end of each block, both the strains and the crack length were experimentally measured at the maximum tensile load. In particular, the crack length is reported in Table 1 as a function of the number of cycles. As already introduced, the target of the proposed methodology is estimating the crack length, which is unknown in a general application; thus, the experimental measurements reported in Table 1 are exploited for validation of the methodology.

### 5.2. iFEM Results with the 3 mm Model

This section systematically investigates all the results obtained with the 3 mm model. In particular, a comprehensive physical description of the likelihood trend is reported in Section 5.2.1, assessing the basic framework of the methodology. Then, the strain field computed by the iFEM is compared with the independent measurements of DIC and the displacement field with laser measurements in Section 5.2.2. Finally, the crack size estimation from multiple measurements at different crack lengths is reported in Section 5.2.3, assessing the capability of the methodology in a real application scenario.

#### 5.2.1. Likelihood Trends

Considering the 3 mm mesh, 9 different models were a priori generated: the undamaged model and 8 models with different crack lengths. Crack lengths span from 6 mm to 48 mm with a step increment of 6 mm, which corresponds to the double of the element’s size, as detailed in Section 4.3.

**Figure 12:**
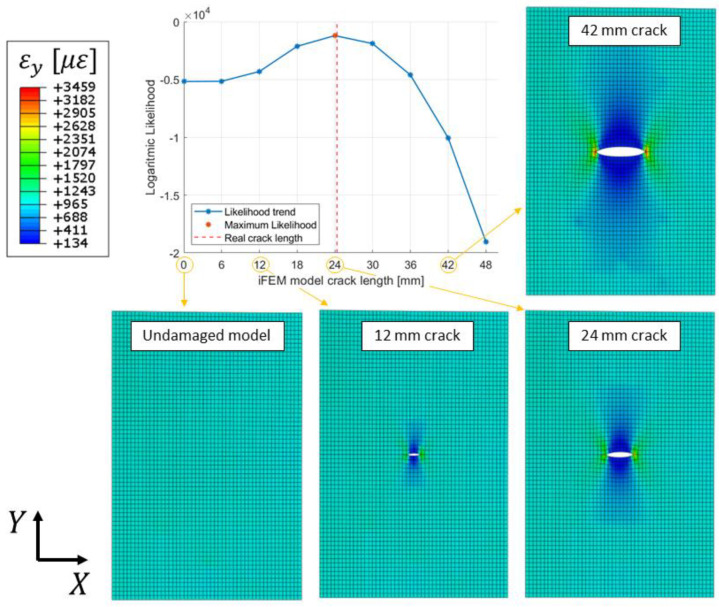
Likelihood trend for experimental strain acquired at a 24.3 mm crack length. Strain field computed by the iFEM for four different models; deformed shape with scale factor 100.

Let us now consider the strain measurements experimentally acquired for a real crack length of 24.3 mm, which was obtained after 20,000 fatigue cycles. The input strain measurements were fed to the 9 iFEM models generated, with each one considering its own physics-based strain pre-extrapolation, as summarized in Figure 5 and Figure 6. Each iFEM model will compute a different strain field, which is in turn compared to the test measurements with the logarithmic Likelihood. The likelihood is computed for a parameter σ=10 με, representing a reasonable standard deviation of the strain’s uncertainties for the present application. Notice that a different value of σ does not affect the results of the present methodology and the maximum likelihood estimation framework would provide the same results since only a scale factor would be applied to its trend. The resulting likelihood trend (Figure 12), which is proportional to the mean square error, resembles the capability of each model of well describing the real damage condition, in particular, the model with the highest logarithmic likelihood considers a 24 mm long crack, which is almost equal to the real one. Finally, models with a different crack length have lower values of likelihood, resembling a probability distribution.

Then, let us analyze in more detail the trend of the likelihood curve obtained. The two iFEM models adjacent to the maximum likelihood point, i.e., the models with 18 and 30 mm cracks, have almost the same likelihood value which is slightly lower than the maximum one. This also indicates that these models have a good probability (The authors naively considered the likelihood function as proportional to the damage probability density function in the presence of a non-informative prior knowledge on damage extent) of describing the real damage condition well; although, this is not the maximum one. Notice that the logarithmic likelihood is used, thus also a small variation of the coefficient magnitude implies a considerable variation agreement between the test and the iFEM strain fields. The same concept also applies to the models with 12 and 36 mm crack lengths; although, they are associated with a significantly lower likelihood. Finally, the likelihood trend exhibits a different behavior for smaller and larger crack lengths, respectively. For large values of crack length (such as 42 and 48 mm) the likelihood exhibits a sudden drop since the test measurements are significantly different from the strains computed by the iFEM. This behavior is induced by the relative distance between the crack tip and the test sensors, which is decreased for larger cracks. On the contrary, a small crack length may induce an almost negligible strain effect in correspondence with the test sensors. For example, the models associated with the undamaged configuration and the 6 mm crack length provide almost the same value of likelihood, since their strain difference is not appreciable in correspondence to the test sensors.

Finally, the strain field computed by iFEM with different models is reported in Figure 12. Notice that the crack’s strain field and the displacement opening of the crack are obtained thanks to the physics-based strain pre-extrapolation technique, which imposes the strain field of each particular crack length on the input formulation, as described in Figure 6. The input measurements acquired by sensors are representative only of the far-field strain; thus, without the physics-based strain pre-extrapolation technique, they would not be able to compute the correct displacement and strain field given by the crack. In particular, if a crack is also present in the model, this would not open since the input strain field is not compatible with the model adopted. The pre-extrapolation technique can be avoided only in cases where all the elements are experimentally covered by input sensors, which is not feasible in a real scenario, in particular in correspondence of a damaged area. In conclusion, the physics-based strain pre-extrapolation technique is fundamental to obtain a displacement and strain field coherent with the crack length imposed in the model. However, all the results obtained by the models considered are potentially wrong since they are associated with a damage condition a priori set by the routine. Thus, among all the results computed, the right model is selected with the maximum likelihood estimation framework, obtaining the most accurate description of the damage state for the given mesh discretization.

**Figure 13:**
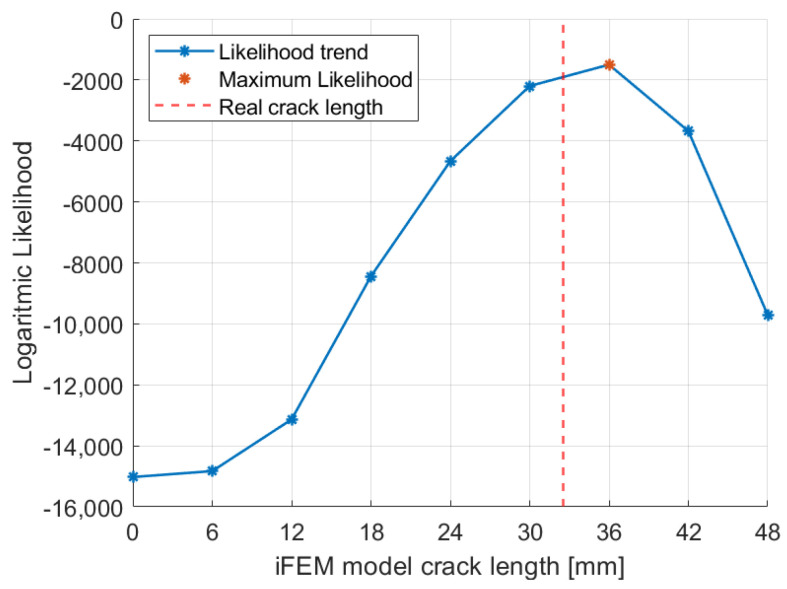
Likelihood trend for experimental strain acquired at 32.5 mm crack length.

The procedure described can be repeated several times for different acquisitions performed in correspondence with different crack lengths. For example, the logarithmic likelihood trend computed for the 32.5 mm crack length strain acquisition, which was obtained after 32,000 fatigue cycles, is reported in Figure 13. In this case, the experimental crack length does not correspond to any of the iFEM models considered and the maximum likelihood estimation framework indicates the 36 mm crack length model as the best. This model considers a crack length immediately after the real one; however, the 30 mm model is also associated with a significant probability.

#### 5.2.2. iFEM Strain and Displacement Fields Evaluation

The strain field computed by the iFEM is compared with the independent measurement of the DIC for a further assessment of the methodology, while the iFEM displacement is compared with the lasers’ measurement.

**Figure 14:**
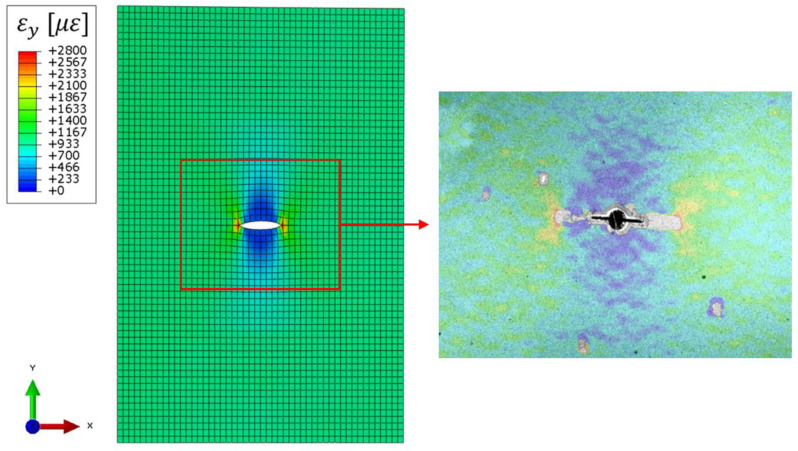
Comparison between iFEM strain reconstruction and the DIC strain field with the same color scale. Acquisition performed at 20,000 cycles: real crack length (DIC) of 24.30 mm and iFEM crack length of 24 mm. Inverse FEM deformed shape with a scale factor 100.

The acquisition performed at 20,000 cycles is considered since the detected crack length by the iFEM (24 mm) is almost equal to the target one (24.30 mm). The strain field along the load direction is compared in Figure 14, showing how the iFEM reconstruction is qualitatively in good agreement with the DIC observation. In particular, the DIC strain field provides a continuous measurement on the whole plate’s area; only the points in proximity of the crack are not computed due to a speckle’s degradation. This provides an independent strain field validation of the whole damage-sensitive area in locations different from the test sensors exploited by the maximum likelihood estimation framework, finally providing the effectiveness of the methodology.

The laser measurements are used to validate the displacement results, in particular the total deformation of the ROI along the load direction. The experimental measurements provide a total deformation between the unloaded and the loaded condition equal to 0.228 mm, while the related iFEM result is equal to 0.207 mm. The laser measurement overestimates about 10% of the iFEM numerical result and this can be attributed to a small misalignment of the lasers, thus measuring a displacement component not perfectly perpendicular to the target surface, and to experimental uncertainties.

After these considerations, the iFEM displacement and strain fields computed with the methodology proposed are accurate and representative of the actual damage condition of the structure. Thus, its adoption in a digital twin framework allows a high-fidelity representation of the structure and can provide precious information about its operative conditions.

#### 5.2.3. Automatic Crack Size Estimation Framework

The crack size estimation procedure is now applied to the whole acquisition history to assess the damage evolution.

**Figure 15:**
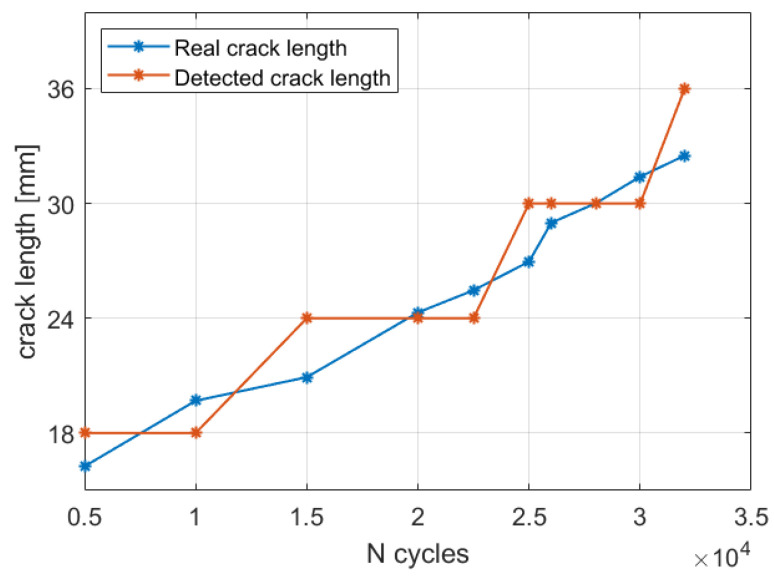
Crack size estimation with the 3 mm iFEM model for the whole acquisition history.

The maximum likelihood estimation framework was applied to each strain acquisition, providing the related diagnosis state. The crack lengths detected by the iFEM were compared with the target measurements in Figure 15. In particular, the detected crack length underestimates or overestimates of the target measurement since only discrete crack’s steps of 6 mm are possible with the present mesh discretization. Nevertheless, the maximum likelihood estimation framework always provides the correct detection, with it giving precious diagnostic information.

However, one main limitation of the proposed technique in a real-time application is the requirement of multiple models with different damage conditions. The iFEM algorithm is particularly attractive for real-time applications since only the strain vector $F_{F}$ of Equation (10) is updated at each iteration, while for a given mesh discretization and sensor network, the matrix $K_{FF}$ is constant and can be inverted just once, resulting in a fast computation of the displacement field. In the present work, an automatic procedure to generate multiple iFEM models has been described (Section 3.2), which can be also exploited to a priori create a database of damage scenarios. However, in a real application, the database cannot consider all the possible damage conditions, since also the damage location is initially unknown. Thus, an attractive procedure is, once the crack center has been identified, generating only the required iFEM models on-demand with the procedure described in Section 3.2. This would allow more accurate damage diagnosis since the model database may not include a damage scenario representative of the real condition. However, each time the mesh is modified by the damage introduction or evolution, the $K_{FF}$ matrix has to be recomputed and reinverted, requiring a significant computational burden. Thus, a compromise has to be found according to the specific application, as summarized by the following approaches:

- The model update and the damage diagnosis are carried out online, where the needed models are generated on-demand, although this may require a significant computation burden
- A database of damage scenarios can be generated offline considering the most relevant damage mechanisms of the structure (e.g., the crack’s nucleation from rivet holes). Then, the real-time algorithm will just load the required models, in which the matrix $K_{FF}$ has been already inverted, and performs an efficient damage diagnosis.
- The damage diagnosis procedure is carried out offline at specified intervals, in parallel with respect to the structure’s operation. With this approach, the a priori definition of the damage scenarios and the database creation is not necessary since the computation does not have strict requirements in terms of computational time. The offline procedure computes the new damage condition of the structure, which can be uploaded on the real-time digital twin and used until the next model’s update. The offline damage diagnosis and model update can be carried out on-demand, for example, based on some real-time damage indices, or at specified intervals according to the expected damage growth rate.
- A combination of the approaches described can be adopted according to the specific case. For example, an initial database can be created, and then, once the damage location is identified, additional models are generated on-demand.

**Figure 16:**
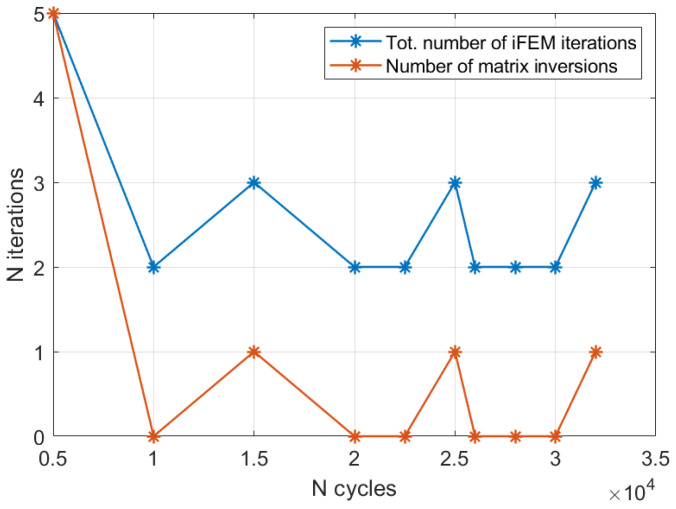
Total number of iFEM iterations and matrix inversions for the whole fatigue propagation.

At this point, regardless of the specific approach adopted, let us suppose to perform the real-time damage diagnosis and digital twin update of the structure under analysis. Let us also suppose that the structure was originally undamaged (thus without any cracks) and after a visual inspection in correspondence of the 5000 cycles acquisition, a crack was discovered and its central position was identified. At this point, the maximum likelihood estimation framework previously described is performed and the crack length detected is 18 mm. In case all the 9 models previously presented are included in the computation, a total of 9 iFEM iterations are required. However, if the computation starts from the undamaged condition and the crack size is gradually increased, the maximum likelihood is correctly identified when the model with the 24 mm damage size is executed, thus at the 5th iteration. More specifically, the damage size is systematically increased until the likelihood trend, after an initial increase, starts decreasing; thus, the selected damage condition is identified by the previous iteration, which is the maximum likelihood estimation. Then, once the crack length in correspondence of the 5000 cycles has been detected, the measurements acquired at 10,000 cycles are processed. The same procedure can be repeated to identify the new crack length; however, the routine can start from the damage size detected at the previous iteration, thus 18 mm in this case. This allows a further reduction in the number of iterations and thus computational time. Moreover, in case the damage scenarios are not loaded from a database, the model computation (i.e., the matrix inversion) of the 18 and 24 mm crack length models is not necessary since it is performed in correspondence to the previous acquisition. Thus, each time a new model is computed for the first time, it can be stored and reused when necessary. This allows a reduction in the computation time during operation since, in case the damage does not significantly propagate, no new iFEM models need to be computed. The acquisition performed at 10,000 cycles will not provide any damage size increase since the likelihood computed by the 24 mm crack size model results are lower than the 18 mm one. Thus, in this specific case, just two iFEM iterations are computed (with two different models) and no new model is generated and inverted, resulting in a very fast computation. The same procedure can be repeated for each iteration and the introduction of a new model, with the related matrix inversion, is carried out only when a crack length propagation is observed, as also summarized in Figure 16. Thus, according to the complexity of the model and the digital twin requirements, the framework proposed can work in real-time without any a priori definition of the damage conditions, generating the required models on-demand. Finally, the numerical procedure described is also detailed by Algorithm 3.

**Algorithm 3:** Real-time crack size estimation procedure without a priori creation of a database of damage scenarios1:Define the undamaged iFEM model (mesh, connectivity, BC, etc.)2:Set the initial state variables:     $iFEM_{model}\left(1\right)$ = undamaged iFEM model (mesh, connectivity, BC, etc.)     $2a_{model}\left(1\right)=0$ (zero initial crack length)     count=0 (time instant counter)     $2a_{detected}\left(0\right)=0$ (zero initial detected crack length)3:Identify the crack center4:Define the input ($\varepsilon_{inp}$) and the test ($\varepsilon_{test}$) sensor networks5:**while** experimental data acquisition ON **do**6:    count=count+1
7:    Acquire the new experimental measurements $\varepsilon_{inp}\left(count\right)$ and $\varepsilon_{test}\left(count\right)$
8:    $index_{model}=$ find ($2a_{model}==2a_{detected}\left(count-1\right))$ (index of the previous iteration iFEM model)9:    iter=1 (iteration counter)10:    ($U\left(iter\right),\;\varepsilon_{iFEM}\left(iter\right)$, LogL(iter)) = compute the iFEM results ($iFEM_{model}$($index_{model})$, $\varepsilon_{inp}\left(count\right)$, $\varepsilon_{test}\left(count\right)$) (Algorithm 4)11:    condition=true
12:    **while** condition==true **do**13:     $index_{model}=$ $index_{model}+1$14:     iter=iter+1
15:     **if** $iFEM_{model}\left(index_{model}\right)$ does not exist **do**16:         **if** $a_{model}\left(index_{model}-1\right)==0$ **do**17:             $iFEM_{model}\left(index_{model}\right)$ = crack introduction from $iFEM_{model}\left(index_{model}-1\right)$ (Algorithm 1)18:         **else**19:             $iFEM_{model}\left(index_{model}\right)$ = crack propagation from $iFEM_{model}\left(index_{model}-1\right)$ (Algorithm 2)20:         **end**21:         Obtain the new $2a_{model}\left(index_{model}\right)$ from $iFEM_{model}\left(index_{model}\right)$
22:     **end**23:     ($U\left(iter\right),\;\varepsilon_{iFEM}\left(iter\right)$, LnL(iter)) = compute the iFEM results ($iFEM_{model}$($index_{model})$, $\varepsilon_{inp}\left(count\right)$, $\varepsilon_{test}\left(count\right)$) (Algorithm 4)24:     **if** LnL(iter−1)>LnL(iter) **do**25:         $2a_{detected}\left(count\right)=2a_{model}\left(index_{model}-1\right)$ (store the detected crack length)26:         $U_{final}\left(count\right)=U\left(iter-1\right)$
27:         $\varepsilon_{iFEM}^{final}\left(count\right)=\varepsilon_{iFEM}\left(iter-1\right)$
28:         condition=false (exit from the while loop)29:     **End**30:    **End**31:
**End**


**Algorithm 4:** iFEM computation subroutine with physics-based strain pre-extrapolation1:Pre-extrapolate the strain field with the physics-based approach considering the $2a_{model}\left(index_{model}\right)$ crack length2:Define the iFEM input vector F
3:Compute the iFEM displacement field $U_{F}$ using Equation (10)4:Compute the iFEM strain field $\varepsilon_{iFEM}$ using Equation (5)5:Compute the logarithmic Likelihood (LnL) between $\varepsilon_{iFEM}$ and $\varepsilon_{test}$ using Equation (12)

### 5.3. iFEM Results with the 1 mm Model

The same methodology is now applied with a finer mesh with a 1 mm element size. The likelihood trends are briefly described in Section 5.3.1, while the final crack size estimation routine is presented in Section 5.3.2.

#### 5.3.1. Likelihood Trends

The 1 mm mesh allows a better discretization of the crack length and so a total number of 25 models are generated. The first model is related to the undamaged structure (no crack), while the other 24 consider different crack lengths up to 48 mm with a step size of 2 mm, corresponding to the double of the element size.

**Figure 17:**
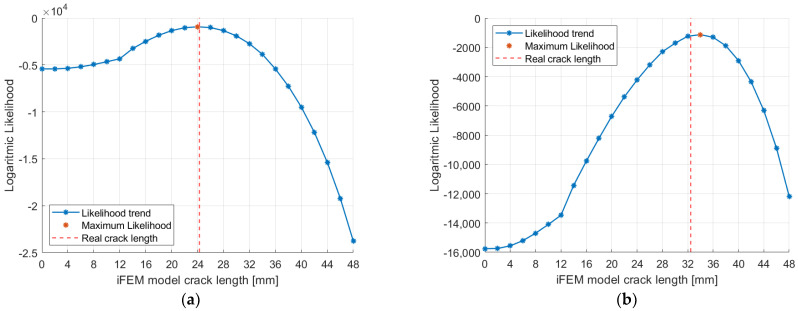
Likelihood trend with the 1 mm model: (**a**) experimental strain acquired at 24.3 mm (20,000 cycles); (**b**) experimental strain acquired at 32.5 mm crack length (32,000 cycles).

The likelihood trends computed for the 20,000 and the 32,000 cycles acquisition are shown in Figure 17 for comparison with respect to Section 5.2.1. In particular, the maximum likelihood is obtained in correspondence of a model that well describes the target damage state, assessing the capability of the technique for a finer mesh. In particular, all the conclusions obtained for the 3 mm model are still valid, but with more accurate detection of the crack length.

#### 5.3.2. Crack Size Estimation Results

**Figure 18:**
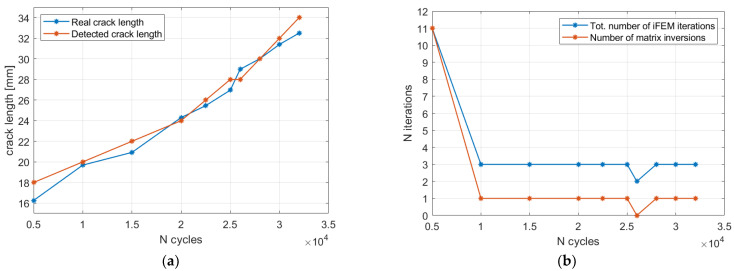
Crack size estimation routine results: (**a**) crack size estimated with respect to the target value of each acquisition; (**b**) the number of iFEM iterations and matrix inversions performed by the real-time algorithm.

The procedure detailed by Algorithm 3 can now be applied to all the acquisitions, with it resembling a real-time application with on-demand generation of the damaged scenarios. The results are reported in Figure 18a, showing a fine discretization of the target crack length. More specifically, the root mean square error between the detected crack length and its true value decreases from 2.05 mm for the 3 mm mesh to 0.97 mm for the 1 mm case. Then, the number of iterations required by the algorithm is shown in Figure 18b. In particular, the computation of a new iFEM model is performed in almost every acquisition since the fine mesh is able to appreciate the damage propagation that has occurred. Obviously, a finer mesh requires a higher computational time for two main reasons: (i) the higher number of elements and degrees of freedom; and (ii) the higher number of model updates required. Thus, in practical applications, a compromise on the mesh size must be chosen and, in that case, the most relevant damage scenarios can be generated a priori and stored in a numerical database to speed up the overall procedure. Another approach could exploit an original coarse mesh defined in the undamaged configuration and then, after the damage is detected and localized, local mesh refinement is performed near the damage affected area. This could also be exploited to generate a fine mesh with elements in a general orientation to detect cracks not modellable with the original mesh discretization. However, this requires a more complex model updating approach than the node duplication described in Section 3.2, and thus it is left to future studies.

## 6. Conclusions

This paper newly extends the load-independent iFEM approach to estimate the structural damage size leveraging on strain measurements acquired on the structure itself.

In case damage occurs on a structure, the iFEM model is no more compatible with the physical structure, inducing a wrong displacement and strain field reconstruction. Thus, the iFEM approach proposed in this work systematically introduces the damage in the iFEM model to decrease this discrepancy. Several iFEM models are generated to account for different possible damage scenarios; thus, considering different damage sizes, then a maximum likelihood estimation framework selects the model that better approaches the real damage condition. This framework minimizes the error between the iFEM strain reconstruction and the measurements from test sensors, which are representative of the real structure’s damaged condition. In addition, the methodology presented overcomes some limitations of previous literature works that were performed on damage detection with the iFEM, in particular: (i) it can account for a lower number of strain sensors; and (ii) it does not require the definition of a threshold for the damage index evaluation.

The methodology was experimentally validated on an aluminum plate subjected to fatigue crack propagation, where the objective of the analysis was the crack length size estimation. The iFEM methodology also exploits the recently developed physics-based strain pre-extrapolation technique to obtain the correct strain gradient induced by the crack and, at the same time, relies on a sparse sensor network constituted of an OBR fiber optic. The methodology proposed provides a crack length estimation very close to the one experimentally measured by visual inspection, although only discrete values are possible according to the mesh discretization adopted. It should be noted that, in case a sufficiently fine mesh is adopted, the minimum difference in the detected crack length loses practical engineering significance. The iFEM strain reconstruction is further validated with the independent observations of DIC, showing how the iFEM is able to well describe the strain field near the crack, even in the presence of a very sparse sensor network.

Nevertheless, the approach proposed has some requirements and limitations which will be further investigated and solved in future works by the authors. As the first main requirement, the damage should be modelled inside the iFEM model, which is relatively easy for the present application on cracks, but it may be non-trivial for other damage mechanisms on composite materials (e.g., delamination), representing an aspect of the methodology to be further investigated. Then, strain sensors should not only describe the global strain field present on the structure but also be sensitive to the presence of damage, thus determining a minimum detectable damage size for a given sensor network. Focusing now on the specific application under analysis, i.e., the crack size estimation, it is assumed that the crack is detected and its location is a priori known from a visual inspection or other identification techniques without accounting for any additional uncertainty on the crack location itself; the joint estimation of the crack location and size is a matter of future research which is currently being developed by the authors. Secondly, the proposed methodology investigates only regular crack growths in which the crack’s direction is constant, while different case studies (e.g., with composite materials) may exhibit different behaviors. In addition, the nucleation or propagation of damage may induce the failure of some sensors, thus representing a loss of input strain measurements, which is still an unexplored aspect in the iFEM literature. The impact of data loss should be systematically evaluated in future studies. Finally, the proposed approach does not require a priori knowledge of the possible damage conditions (i.e., of the possible crack positions and lengths) since the required models are generated on-demand by the algorithm itself, which is beneficial compared to other diagnosis approaches, e.g., based on machine learning techniques, where the a priori generation of different damage conditions (i.e., a database of damage scenarios) is a fundamental step. However, each time a new damage condition is generated, the iFEM matrix $K_{FF}$ should be computed and inverted, resulting in a computationally demanding task. This point is the most significant limitation of the present technique and, in view of realizing a computationally efficient algorithm that can work in real-time applications also for complex structures, different strategies can be adopted. For example, the most relevant damage scenarios can be a priori generated and then specific models, more representative of the actual damage condition, can be computed on-demand. As an alternative, model updating can be run simultaneously to structure operation and, once the most likely model is selected, it will be provided as input to the real-time routine. All the hypotheses and limitations are summarized in Table 2 for completeness.

These approaches, opportunely combined, can result in a fast routine that can be exploited to develop an iFEM based digital twin (iFEM-DT). This is a high-fidelity model of the real structure, which is constantly updated according to the damage evolution, maintenance operations, and other structure modification. Thus, the automatic damage size estimation based on iFEM proposed in this work appears to be a good candidate to develop new DT frameworks.

In conclusion, this work introduces the possibility to include the damage information in the iFEM model, which can be exploited in an SHM or a DT framework. The future works of the authors will be dedicated to further developing the proposed methodology and assessing its capabilities in different, more complex scenarios.

## Figures and Tables

**Table 1:** Experimental target crack length measured at different fatigue load cycles.

N Cycles	Crack Length [mm]
5000	16.26
10,000	19.69
15,000	20.92
20,000	24.30
22,500	25.46
25,000	26.96
26,000	28.99
28,000	30.01
30,000	31.40
32,000	32.50

**Table 2:** Main hypotheses and drawbacks of the iFEM model updating procedure proposed for crack size estimation, together with future developments to meet some hypotheses.

Hypotheses	Drawbacks	Future Activities
The damage must be modelled in the iFEM model. The damage location is assumed to be known. Accurate description of the input strain field, if necessary leveraging on the strain pre-extrapolation technique. Test sensors must be sensitive to the presence of damage. Straight crack propagation with constant principal stresses in time.	Higher computational time required for: (i) displacement calculation from multiple iFEM models; and (ii) matrix inversion of the iFEM models (if necessary). Necessity to a priori generate some damage scenarios to decrease the computational time.	Development of a joint estimation of the crack location, orientation, and size. Implementation of a local mesh refinement to account for cracks with an arbitrary orientation.

## Data Availability

Data available on reasonable request to the authors.
